# Effect of Non-surgical Periodontal Therapy on the Salivary Resistin Level in Obese and Non-obese Individuals With Periodontitis

**DOI:** 10.7759/cureus.100157

**Published:** 2025-12-26

**Authors:** Shijna Ashraf, S. Santhosh Kumar, Rekha P Radhakrishnan, R M Baiju, Nebu George, Neethu P Reghu, Tony Kurien, Jeswin Johnson

**Affiliations:** 1 Department of Periodontics, Government Dental College Kottayam, Kottayam, IND; 2 Department of Periodontics, Pushpagiri College of Dental Sciences, Thiruvalla, IND

**Keywords:** obesity, periodontal therapy, periodontitis, resistin, saliva

## Abstract

Background

The bidirectional relationship between obesity and periodontitis has been well-established, and adipokines play a key role in this association. Among adipokines, salivary resistin has been relatively less studied as a potential marker of systemic inflammation in obese individuals with periodontitis. With the increasing burden of lifestyle diseases, understanding the effects of periodontal therapy on the systemic inflammatory status of obese individuals will aid in more comprehensive health management. Therefore, this study aimed to compare salivary resistin levels and periodontal parameters before and 12 weeks after non-surgical periodontal therapy (NSPT) in obese and non-obese individuals with generalized stage II grade B periodontitis.

Methods

This longitudinal study included 60 participants with periodontitis. Participants were divided into two groups based on body mass index (BMI): the obese group (Group I) with BMI ≥ 27.5 kg/m² and the non-obese group (Group II) with BMI ≤ 27.5 kg/m². At baseline, salivary resistin levels and periodontal parameters, including probing pocket depth (PPD), clinical attachment level (CAL), full mouth plaque score (FMPS), and full mouth bleeding score (FMBS), were assessed. All participants underwent NSPT, with no local or systemic antibiotics or adjunctive treatments. Salivary resistin levels and periodontal parameters were reassessed 12 weeks post-treatment.

Results

Intergroup comparisons revealed higher resistin levels and periodontal parameters in the obese group at baseline and at the 12-week follow-up. Both groups showed a significant reduction in salivary resistin levels and clinical parameters after NSPT (p value<0.05). Even though the obese group demonstrated a greater magnitude of improvement in several measures, including salivary resistin, FMPS and FMBS after NSPT (p value<0.05), the values remain high compared to the non-obese group. Moreover, the non-obese group demonstrated a significant reduction in THE PPD and CAL indicating a pronounced clinical recovery following NSPT (p value<0.05).

Conclusion

Obesity contributes significantly to the severity of periodontitis by enhancing systemic inflammatory burden. NSPT effectively improved clinical periodontal health and reduced salivary resistin levels in individuals with periodontitis, regardless of obesity status. The obese group demonstrated more significant changes in salivary resistin levels following NSPT, but the clinical recovery in terms of PPD and CAL was better for the non-obese group. This study underscores the importance of integrating personalized periodontal care in obese individuals, along with other strategies aimed at reducing obesity associated inflammation to optimize long-term health outcomes.

## Introduction

Obesity is characterized by excessive accumulation of body fat relative to lean body mass, to the extent that it impairs health [1]. Body mass index (BMI) is a globally accepted metric for assessing obesity and is calculated by dividing a person’s weight in kilograms by the square of their height in meters (kg/m²) [2]. A BMI > 25 kg/m² is considered overweight, and BMI > 30 kg/m² is classified as obesity in adults [3]. In younger and middle-aged individuals, mortality risk increases consistently with increasing BMI. However, this relationship becomes less straightforward in older adults, highlighting the need for additional measures of body composition to more accurately assess mortality risk [4].

A recent systematic review by Trindade et al. (2023) demonstrated a notably higher current prevalence of periodontitis in dentate individuals compared with that in the preceding decade [5]. This represents a major public health concern, as epidemiological evidence links periodontitis with several systemic diseases, including diabetes mellitus, rheumatoid arthritis, cardiovascular diseases, and obesity [6].

The relationship between obesity and periodontitis represents an important area of investigation in periodontal medicine. Saito et al. first reported this association in humans in 1998 [7]. The precise biological mechanisms linking periodontitis and obesity are not yet fully elucidated. Adipose tissue secretes proinflammatory cytokines and hormones, collectively termed adipocytokines or adipokines, which contribute to systemic inflammation and oxidative stress, potentially linking the two conditions [8].

Resistin is a hormone-like protein or adipokine, first identified in mice in 2001 and named for its ability to resist insulin action [9]. In mice, it is mainly secreted by adipose tissue and certain immune cells such as macrophages [9]. Human resistin is a cysteine-rich peptide weighing approximately 12.5 kDa and is mainly secreted by peripheral blood mononuclear cells, macrophages, and bone marrow cells, rather than by adipose tissue [9,10]. The normal serum concentration of resistin is 7-22 ng/mL, but it is elevated in individuals with diabetes mellitus, atherosclerosis, inflammatory bowel disease, obesity, periodontitis, and several other systemic diseases [9-11]. Resistin is now recognized as an important biomarker and a potential therapeutic target in numerous diseases.

Human saliva is an important diagnostic fluid and reflects the overall health of the individual. Saliva can be collected noninvasively without specialized skills or equipment and is ideal for large-scale population screening. Resistin has been detected in saliva and shows a positive correlation with serum resistin [12,13].

Although the association between obesity and periodontitis has been well established, few studies have investigated whether periodontal therapy influences systemic inflammation differently in obese and non-obese individuals. Therefore, this study aimed to evaluate the effect of non-surgical periodontal therapy (NSPT) on salivary resistin levels in obese and non-obese individuals with generalized stage II grade B periodontitis, to emphasize the importance of oral healthcare in obese individuals.

## Materials and methods

Study design

The longitudinal clinical study was conducted in the Outpatient Department of Periodontics, Government Dental College, Kottayam, India over a period of one year from August 1, 2023 to July 31, 2024. The study was conducted in accordance with the ethical principles of Declaration of Helsinki, as revised in 2013. The Institutional Ethical Committee approved the study protocol (Approval number: IEC/M25/2023/R466/DCK dated 23-05-2023). All procedures were performed by a single principal investigator.

Sample size calculation

The sample size was calculated based on the observations from a similar study by Al-Hamoudi et al. [14], applying the following formula:

 "$&quot; N=\frac{\frac{r+1}{r}\ \times\ {SD}^2\ \times\left(\frac{Z\alpha}{2}+Z\beta\right)^2}{d^2} &quot;$"

where r = ratio of cases to controls; Zβ = standard normal variate at 80% power = 0.84; Zα/2 = standard normal variate at 5% type 1 error = 1.96; d = mean difference between the groups =1.2; and SD = pooled standard deviation =1.595. Substituting the values, we obtained sample size N = 27.7, which was rounded to 30 per group. A consecutive sampling method was employed until the desired sample size was attained. The inclusion and exclusion criteria adopted for the selection of study participants are presented in Table 1.

**Table 1:** Inclusion and exclusion criteria applied in the selection of study participants CAL, clinical attachment level.

Inclusion Criteria	Exclusion Criteria
Adults > 18 years of age	Pregnant or lactating women
Diagnosed with periodontitis. (Periodontitis is defined as interdental CAL ≥ 2 mm in ≥ two non-adjacent teeth or buccal CAL ≥ 3 mm with probing depth > 3 mm in ≥ two teeth, with CAL not attributable to non-periodontitis related causes)	Antibiotic therapy within the previous three months
	Periodontal treatment within the previous six months
	Intellectual disability that could interfere with oral hygiene procedures
	Unwilling to provide consent for the study
	Smokers (≥ 10 cigarettes/day)
	Individuals diagnosed with systemic diseases such as uncontrolled diabetes mellitus, rheumatoid arthritis, cardiovascular diseases, and inflammatory bowel disease.

Data collection

After applying the inclusion and exclusion criteria, eligible participants were provided a detailed explanation about the study procedures and enrolled after obtaining written informed consent. Demographic data, height, weight, and BMI were recorded. Participants were divided into two groups based on BMI cutoff value of 27.5 kg/m², reflecting the higher metabolic risk threshold for Asian populations [15]: (1) Obese group (Group I, cases with exposure-periodontitis patients with obesity, BMI ≥ 27.5 kg/m^2^) and (2) Non-obese group (Group II-periodontitis patients without obesity, BMI ≤ 27.5 kg/m^2^).

Saliva collection

Unstimulated whole saliva (2 ml) was collected from all participants for the assessment of salivary resistin levels. Morning saliva samples were collected between 8:00 and 9:00 AM following an overnight fast. Participants were comfortably seated on a chair, with their heads bent slightly forward. They were instructed to allow saliva to pool in the mouth in the absence of any oral movements and then expectorate the saliva into a collection tube. Samples were refrigerated within 30 minutes and subsequently transported to the test center (Pushpagiri Research Centre, Thiruvalla, Kerala, India). They were stored at ˗80°C within four hours of sample collection until further analysis. Salivary resistin levels were measured using a human RETN (Resistin) enzyme-linked immunosorbent assay (ELISA) kit, Cat: ITLK01225 with a sensitivity specification 0.056 ng/mL and detection range 0.16-10 ng/mL (G-Biosciences, St. Louis, MO, USA).

ELISA test procedure

The principle of this kit is based on a sandwich enzyme immunoassay. The microtiter plate provided is pre-coated with an antibody specific for resistin (RETN). Standards or samples are added to the appropriate wells, followed by a biotin-conjugated antibody specific to RETN. Next, avidin conjugated to horseradish peroxidase (HRP) is added to each well and incubated. After incubation, 3,3’,5,5’-tetramethylbenzidine (TMB) substrate solution is added; only wells containing RETN, the biotin-conjugated antibody, and enzyme-conjugated avidin will exhibit a color change. The enzyme-substrate reaction is stopped by the addition of stop solution, and the resulting color change is measured spectrophotometrically at 450 nm ± 10 nm. The concentration of RETN in the samples is then determined by comparing the optical density (OD) of the samples to the standard curve. The test procedure was performed by single well-trained laboratory personnel to minimize measurement variability.

Clinical parameters

After saliva collection, the following clinical parameters were recorded on the same day by a calibrated single examiner to minimize the measurement bias:

Probing Pocket Depth (PPD) and CAL

The PPD is measured as the distance from the base of the pocket to the gingival margin using a University of North Carolina-15 probe (UNC-15 probe; Hu-Friedy® Manufacturing Inc., Chicago, IL, USA) by inserting the probe parallel to the long axis of the teeth. CAL is measured as the distance from cementoenamel junction (CEJ) to the base of the pocket using a UNC-15 probe. A standard probing force of 20 grams which lightly blanches the fingernail used to assess the periodontal parameters. Measurements were recorded at six sites per tooth including the third molars. The mean PPD and mean CAL for each patient were calculated.

Full Mouth Plaque Score (FMPS) and Full Mouth Bleeding Score (FMBS)

The presence or absence of plaque (O’Leary’s plaque index [16]) and bleeding were recorded. Scores were assigned as 0 (absent) or 1 (present). Measurements were made at four sites per tooth, and the sum total of scores from all surfaces with plaque and bleeding was divided by the sum total of all surfaces separately.

Intervention and follow-up

A thorough NSPT (Steps 1 and 2) was performed for all participants on two different appointments within one week under local anesthesia using standard Gracey curettes (Hu-Friedy, Chicago, IL, USA) and ultrasonic scalers (Model UDS-J, Guilin Woodpecker Medical Instrument Co., Ltd., Guilin, China). They were instructed to strictly follow the oral hygiene instructions provided. Participants were recalled after 12 weeks, and saliva samples were collected again to assess post-treatment resistin levels. Post-treatment clinical parameters were also recorded.

Statistical analysis

The collected data were entered into Microsoft® Excel® 2019 (Version 2403 Build 16.0.17425.20176; 64-bit; Microsoft Corporation, Redmond, USA) and subsequently exported to IBM SPSS Statistics for Windows, Version 29 (Released 2024; IBM Corp., Armonk, New York, United States) for statistical analysis.

The Shapiro-Wilk test was used to assess the distribution of data in both groups. Most parameters in the obese and non-obese groups demonstrated non-normal distribution, particularly FMBS, FMPS, PPD, and CAL at baseline and follow-up. Therefore, non-parametric tests, such as the Wilcoxon signed-rank test and Mann-Whitney U test, were used for most comparisons. The level of significance was set at 5% (p-value < 0.05) with a 95% confidence interval.

## Results

Baseline characteristics such as age, gender distribution, BMI, and smoking status were recorded and analyzed (Table 2). Although the proportion of smokers was slightly higher in the obese group, the overall prevalence was low, minimizing its potential influence as a confounding factor in the observed outcomes.

**Table 2:** Comparison of demographic characteristics of the participants BMI, body mass index

Parameter		Obese Group	Non-obese Group
Age (Mean ± SD) (years)		35 ± 8.5	33.9 ± 7.5
Gender (N%)	Male	16 (53.3%)	17 (56.7%)
	Female	14 (46.7%)	13 (43.3%)
BMI (Mean ± SD) (kg/m^2^)		31.892 ± 2.8335	21.132 ± 1.979
Smoking status (N%)	Yes	5 (16.7%)	2 (6.7%)
	No	25 (83.3%)	28 (93.3%)

Compared to the baseline values, at 12 weeks, the obese group showed significant reduction in all parameters, including resistin levels, FMBS, FMPS, PPD, and CAL, indicating a positive response to periodontal treatment in obese patients (Table 3).

**Table 3:** Comparison of resistin level, FMBS, FMPS, PPD, and CAL at baseline and follow-up in the obese group (Group I) p < 0.05 was considered significant (Wilcoxon signed-rank test). IQR, interquartile range; FMBS, full mouth bleeding score; FMPS, full mouth plaque score; PPD, probing pocket depth; CAL, clinical attachment level.

Variable	N	Baseline Median (IQR)	12 weeks Median (IQR)	p-value
Resistin level (ng/mL)	30	6.43 (5.78–7.23)	6.07 (5.33–7.03)	0.001
FMBS (%)	30	86 (85–95)	33 (26–36)	0.001
FMPS (%)	30	89 (87.75–95.25)	30 (25.75–32.75)	0.001
PPD (mm)	30	4.50 (4.34–4.67)	3.19 (3.06–3.40)	0.001
CAL (mm)	30	4.51 (4.36–4.72)	3.22 (3.08–3.46)	0.001

Non-obese participants also presented similar significant improvements in resistin levels and clinical periodontal parameters at 12 weeks (Table 4).

**Table 4:** Comparison of resistin level, FMBS, FMPS, PPD, and CAL at baseline and follow-up in the non-obese group (Group II) p < 0.05 was considered significant (Wilcoxon signed-rank test). IQR, interquartile range; FMBS, full mouth bleeding score; FMPS, full mouth plaque score; PPD, probing pocket depth; CAL, clinical attachment level.

Variable	N	Baseline Median (IQR)	12 Weeks Median (IQR)	p-value
Resistin level (ng/mL)	30	4.12 (3.45–4.26)	3.90 (3.21–4.02)	0.001
FMBS (%)	30	67.50 (61.5–76)	25 (24–26)	0.001
FMPS (%)	30	69 (67.7–80)	26.5 (24–27)	0.001
PPD (mm)	30	4.12 (3.96–4.18)	2.35 (2.17–2.46)	0.001
CAL (mm)	30	4.13 (4.03–4.23)	2.37 (2.22–2.48)	0.001

**Figure 1:**
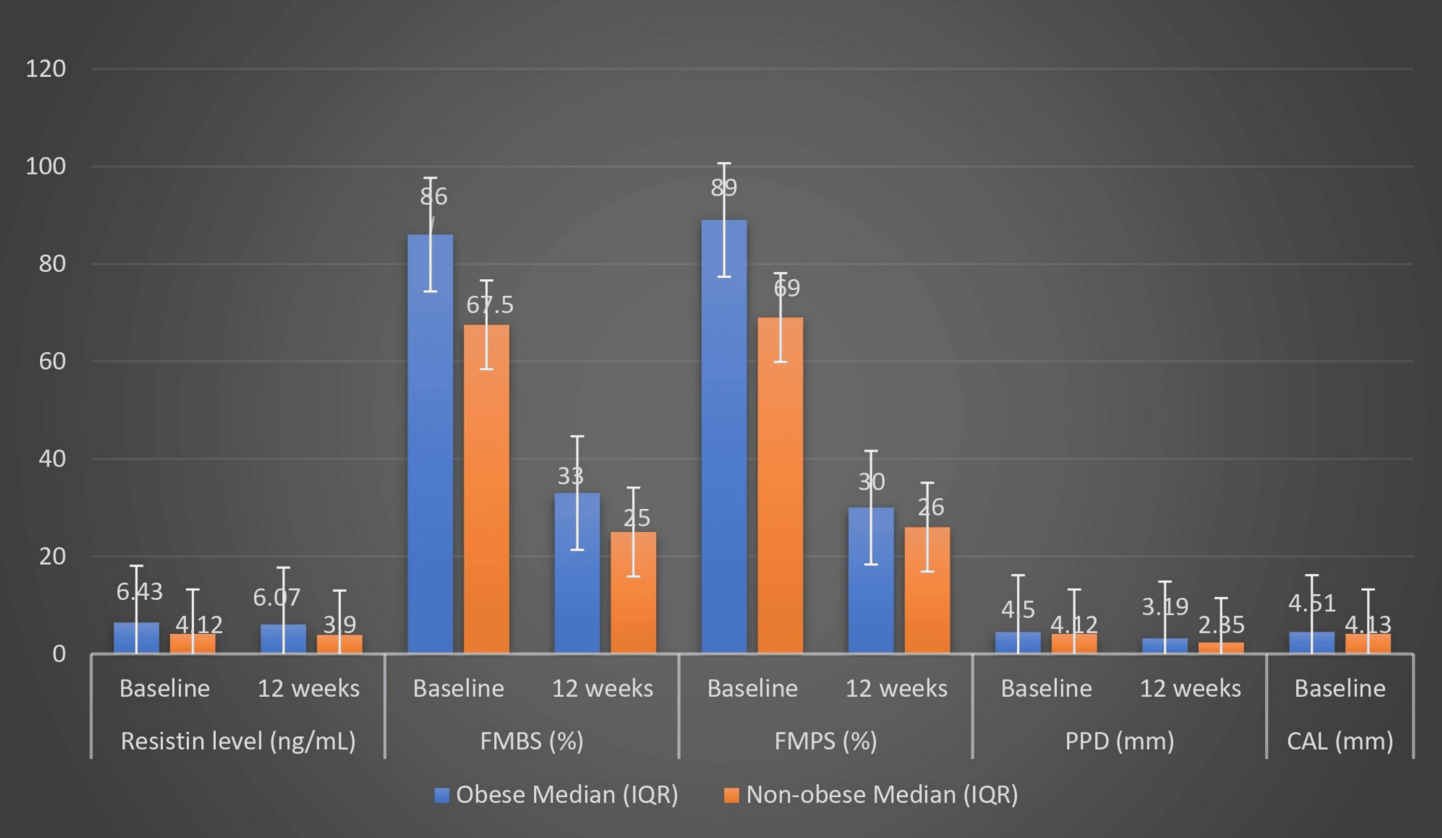
Comparison of resistin, FMBS, FMPS, PPD, and CAL levels at baseline and follow-up between the groups FMBS, full mouth bleeding score; FMPS, full mouth plaque score; PPD, probing pocket depth; CAL, clinical attachment level.

At baseline, participants with obesity had significantly higher median resistin levels (6.43 ng/mL; interquartile range (IQR): 5.78-7.20) than participants without non-obesity (4.12 ng/mL; IQR: 3.45-4.26; p = 0.001). Similarly, periodontal indices also indicated a more severe disease status in the obese group at baseline. At 12 weeks, despite improvements in both groups, the obese group had significantly higher median resistin levels (6.07 ng/ml; IQR: 5.33-7.03) compared with the non-obese group (3.90 ng/ml; IQR: 3.21-4.02; p = 0.001). As with the baseline, the obese group demonstrated more severe disease status at 12 weeks (Table 5; Figure 1).

**Table 5:** Comparison of resistin level, FMBS, FMPS, PPD, and CAL at baseline and 12-week follow-up between the groups p < 0.05 was considered significant (Mann–Whitney U test). FMBS, full mouth bleeding score; FMPS, full mouth plaque score; PPD, probing pocket depth; CAL, clinical attachment level.

Variable	N		Obese Median (IQR)	Non-obese Median (IQR)	p-value
Resistin level (ng/mL)	60	Baseline	6.43 (5.78–7.2)	4.12 (3.45–4.26)	0.001
		12 weeks	6.07 (5.33–7.03)	3.90 (3.21–4.02)	0.001
FMBS (%)	60	Baseline	86 (85–95)	67.5 (61.5–76.0)	0.001
		12 weeks	33 (26–36)	25 (24–26)	0.001
FMPS (%)	60	Baseline	89 (87.7–95.2)	69 (67.7–80)	0.001
		12 weeks	30 (25.7–32.7)	26 (24–27)	0.001
PPD (mm)	60	Baseline	4.5 (4.36–4.72)	4.12 (3.96–4.18)	0.001
		12 weeks	3.19 (3.06–3.4)	2.35 (2.17–2.46)	0.001
CAL (mm)	60	Baseline	4.51 (4.36–4.72)	4.13(4.03–4.23)	0.001
		12 weeks	3.22 (3.08–3.46)	2.37 (2.22–2.37)	0.001

The magnitude of change in all parameters from baseline to 12 weeks was also assessed. The obese group had a higher median change in resistin levels (0.25 ng/mL Vs. 0.22 ng/mL), FMBS (55 % Vs. 44.5 %), and FMPS (60 % Vs. 46.5 %) compared to the non-obese group. Reduction in the PPD (1.73 mm Vs. 1.26 mm) and CAL (1.75 mm Vs. 1.26 mm) was more marked in the non-obese group than in the obese group. All differences were significant (p = 0.001), suggesting that although both groups benefited from NSPT, non-obese individuals experienced slightly more pronounced clinical recovery (Table 6).

**Table 6:** Comparison of change in the resistin level, PPD, CAL, FMBS, and FMPS from the baseline to 12 weeks p < 0.05 was considered significant (Mann–Whitney U test), FMBS, full mouth bleeding score; FMPS, full mouth plaque score; PPD, probing pocket depth; CAL, clinical attachment level.

Variable	N	Obese Median (IQR)	Non-obese Median (IQR)	P value
Change in resistin level (ng/mL)	60	0.25 (0.17–0.40)	0.22 (0.11–0.30)	0.001
Change in FMBS (%)	60	55 (49–62)	44.5 (35–51.25)	<0.001
Change in FMPS (%)	60	60 (55.7–65)	46.5 (39.5–53)	0.001
Change in PPD (mm)	60	1.26 (1.16–1.57)	1.73 (1.53–1.87)	0.001
Change in CAL (mm)	60	1.26 (1.16–1.57)	1.75 (1.53–1.87)	0.001

## Discussion

This longitudinal clinical study evaluated the impact of NSPT on salivary resistin levels, with a particular focus on the significance of monitoring post-treatment resistin levels in obese individuals. Obesity alone does not appear to induce pathological periodontal alterations in a healthy oral environment. However, animal studies indicated that the contribution of obesity to periodontal destruction significantly increases in the presence of local irritants or bacterial plaque [17]. This observation led to further investigations into the obesity-periodontitis relationship and Saito et al. in 1998 first reported a positive association between obesity and periodontitis in Japanese adults, showing that individuals with higher BMI had deeper periodontal pockets and greater attachment loss [7]. The distribution of body fat also plays an important role, as individuals with visceral obesity exhibit more pronounced periodontal destruction [18]. Genco et al. analyzed data from the Third National Health and Nutrition Examination Survey (NHANES III) and demonstrated that BMI was positively correlated with the severity of periodontal attachment loss [19]. Moreover, several systematic reviews highlight the positive association between obesity and periodontitis [20,21].

The exact molecular mechanisms linking obesity and periodontitis are yet to be elucidated. In obese individuals, adipose tissue and adipose-tissue macrophages secrete elevated levels of adipokines, such as leptin, resistin, visfatin, and chemerin, contributing to systemic inflammation [22]. Moreover, obesity is associated with increased production of TNF-α and elevated expression of its soluble receptors [19]. This persistent pro-inflammatory state, combined with oxidative stress, induces insulin resistance, thereby modulating the relationship of obesity with several conditions, such as diabetes and periodontitis [20,23].

Since the discovery of resistin by Steppan et al. in 2001, studies have suggested that resistin may act as a molecular link between diabetes, obesity, and periodontitis [9,11]. The systemic pro-inflammatory state in obese individuals may lead to increased concentration of cytokines in the gingival crevicular fluid, which has been speculated as a reason for the association between obesity and periodontitis. However, a systematic review and meta-analysis by Akram et al. found inconclusive evidence supporting elevated levels of proinflammatory biomarkers, including resistin, in gingival crevicular fluid [24].

Given the limited evidence on the effect of NSPT on salivary resistin levels, this study was undertaken to further explore this relationship. At baseline, the obese group exhibited significantly higher levels of salivary resistin compared to the non-obese group, which aligns with findings from previous studies by Rao et al., Akram et al., and Suresh et al. [13,25,26]. This finding also supports the evidence that obesity is associated with chronic low-grade inflammation and increased circulating adipokines. Following NSPT, salivary resistin levels decreased significantly in both groups in our study, indicating that the benefits of NSPT extend beyond local periodontal tissues and may contribute to systemic anti-inflammatory effects. Similar reductions in salivary and serum resistin levels following NSPT were noted in several previous studies by Al-Hamoudi et al., Akram et al., and Suresh et al. [14,25,26]. In this study, obese individuals exhibited a significantly greater reduction in resistin levels following NSPT compared to non-obese individuals, reflecting the impact of nonsurgical periodontal therapy in reducing the systemic inflammatory burden. But still the 12 weeks postoperative resistin value remains high for the obese group. This supports the hypothesis proposed by Kantarci et al. that periodontal therapy alone may not fully counteract the inflammatory burden in individuals with obesity, and a more comprehensive management, including weight reduction and systemic health monitoring, may be required to achieve optimal outcomes [27]. Our findings contrast with those of Suresh et al., who reported greater reduction in serum resistin levels in normal-weight individuals [26]. In contrast to our finding, Goncalves et al. reported no changes in adipokine levels after scaling and root planing [28,29]. Similarly, Devanoorkar et al. observed only an insignificant change in serum resistin levels following NSPT [30].

Although NSPT resulted in improvements in the periodontal parameters after 12 weeks in both groups, the improvement in PPD and CAL was less pronounced in the obese group. This finding aligns with the conclusions of a systematic review by Gerber et al. that obesity may be associated with poor outcomes after periodontal therapy [31].

Most previous studies assessed serum resistin, whereas in this study, saliva was used as the diagnostic sample because of the following reasons. Saliva is rich in proteins and genetic material, and collection of saliva is cost-effective, easy, and patient-friendly. Moreover, studies have shown a positive correlation between salivary and serum resistin levels [12,13]. There is a lack of population-based studies that use saliva to assess resistin levels following periodontal therapy in obese individuals, and the present study helps address this gap, particularly in this specific population.

The present study possesses several strengths that enhance the validity and reliability of its findings. In the present study, both groups were well matched for age and gender, minimizing the risk of age and gender-related confounding, as these variables can independently affect periodontal disease severity and tissue healing after periodontal therapy. Other baseline characteristics were also comparable between the obese and non-obese groups. Comprehensive assessment of clinical periodontal parameters (PPD, CAL, FMBS, and FMPS) and salivary resistin level provided valuable insights into the relationship between obesity and periodontal health. The use of saliva, a non-invasive diagnostic fluid, further strengthened the study design. The observed reduction in salivary resistin levels following NSPT supports its potential as a biomarker for monitoring systemic inflammatory responses to periodontal therapy. Consistent and significant improvements across all assessed parameters in both groups confirm the effectiveness of NSPT, while persistent intergroup differences signify the influence of obesity-related systemic inflammation on periodontal health.

The study had some limitations, including a short follow-up duration and a small sample size. Additionally, resistin was the only inflammatory marker assessed in this study. We acknowledge potential confounding from low‑level smoking in participants, ELISA kit assay variability, and single‑center design as additional limitations. Future randomized controlled studies with longer follow-up are required for more relevant interpretations.

## Conclusions

Measuring resistin levels in saliva offers an effective method to evaluate the impact of NSPT on obese individuals with periodontitis. NSPT effectively improved periodontal health and lowered salivary resistin levels in individuals with periodontitis, regardless of obesity status. Although both obese and non-obese individuals experienced improvements, the non-obese group demonstrated lower values of resistin and periodontal parameters at 12 weeks. This finding highlights the influence of obesity as a contributing factor in impairing periodontal healing and treatment outcomes, likely due to persistent systemic inflammation associated with increased adipokine secretion from adipose tissue.

From a clinical perspective, this study reinforces the importance of a personalized treatment approach for periodontitis patients with obesity. Although conventional NSPT yields significant improvements in obese patients, its benefits may be enhanced when combined with weight reduction, nutritional counseling, and lifestyle modifications as part of an integrated treatment approach.

## References

[1] Klauer J, Aronne LJ (2002). Managing overweight and obesity in women. Clin Obstet Gynecol.

[2] Blackburn H, Jacobs D Jr (2014). Commentary: origins and evolution of body mass index (BMI): continuing saga. Int J Epidemiol.

[3] (2000). Obesity: preventing and managing the global epidemic. Report of a WHO consultation. World Health Organ Tech Rep Ser.

[4] Freedman DM, Ron E, Ballard-Barbash R, Doody MM, Linet MS (2006). Body mass index and all-cause mortality in a nationwide US cohort. Int J Obes (Lond).

[5] Trindade D, Carvalho R, Machado V, Chambrone L, Mendes JJ, Botelho J (2023). Prevalence of periodontitis in dentate people between 2011 and 2020: a systematic review and meta-analysis of epidemiological studies. J Clin Periodontol.

[6] Nascimento GG, Leite FR, Correa MB, Horta BL, Peres MA, Demarco FF (2014). Relationship between periodontal disease and obesity: the role of life-course events. Braz Dent J.

[7] Saito T, Shimazaki Y, Sakamoto M (1998). Obesity and periodontitis. N Engl J Med.

[8] Jagannathachary S, Kamaraj D (2010). Obesity and periodontal disease. J Indian Soc Periodontol.

[9] Steppan CM, Bailey ST, Bhat S (2001). The hormone resistin links obesity to diabetes. Nature.

[10] Jamaluddin MS, Weakley SM, Yao Q, Chen C (2012). Resistin: functional roles and therapeutic considerations for cardiovascular disease. Br J Pharmacol.

[11] Devanoorkar A, Kathariya R, Guttiganur N, Gopalakrishnan D, Bagchi P (2014). Resistin: a potential biomarker for periodontitis influenced diabetes mellitus and diabetes induced periodontitis. Dis Markers.

[12] Yin J, Gao H, Yang J, Xu L, Li M (2012). Measurement of salivary resistin level in patients with type 2 diabetes. Int J Endocrinol.

[13] Rao RM, Shenoy N, Thomas B (2016). Estimation of serum and salivary level of resistin in obese patients with periodontitis. Indian J Oral Sci.

[14] Al-Hamoudi N, Abduljabbar T, Mirza S, Al-Sowygh ZH, Vohra F, Javed F, Akram Z (2018). Non-surgical periodontal therapy reduces salivary adipocytokines in chronic periodontitis patients with and without obesity. J Investig Clin Dent.

[15] (2004). Appropriate body-mass index for Asian populations and its implications for policy and intervention strategies. Lancet.

[16] O'Leary TJ, Drake RB, Naylor JE (1972). The plaque control record. J Periodontol.

[17] Perlstein MI, Bissada NF (1977). Influence of obesity and hypertension on the severity of periodontitis in rats. Oral Surg Oral Med Oral Pathol.

[18] Saito T, Shimazaki Y, Koga T, Tsuzuki M, Ohshima A (2001). Relationship between upper body obesity and periodontitis. J Dent Res.

[19] Genco RJ, Grossi SG, Ho A, Nishimura F, Murayama Y (2005). A proposed model linking inflammation to obesity, diabetes, and periodontal infections. J Periodontol.

[20] Martinez-Herrera M, Silvestre-Rangil J, Silvestre FJ (2017). Association between obesity and periodontal disease. A systematic review of epidemiological studies and controlled clinical trials. Med Oral Patol Oral Cir Bucal.

[21] Kim CM, Lee S, Hwang W, Son E, Kim TW, Kim K, Kim YH (2022). Obesity and periodontitis: a systematic review and updated meta-analysis. Front Endocrinol (Lausanne).

[22] Taylor EB (2021). The complex role of adipokines in obesity, inflammation, and autoimmunity. Clin Sci (Lond).

[23] Abdalla MM (2021). Salivary resistin level and its association with insulin resistance in obese individuals. World J Diabetes.

[24] Akram Z, Abduljabbar T, Abu Hassan MI, Javed F, Vohra F (2016). Cytokine profile in chronic periodontitis patients with and without obesity: a systematic review and meta-analysis. Dis Markers.

[25] Akram Z, Baharuddin NA, Vaithilingam RD (2017). Effect of nonsurgical periodontal treatment on clinical periodontal variables and salivary resistin levels in obese Asians. J Oral Sci.

[26] Suresh S, Mahendra J, Singh G, Pradeep Kumar AR, Thilagar S, Rao N (2018). Effect of nonsurgical periodontal therapy on plasma-reactive oxygen metabolite and gingival crevicular fluid resistin and serum resistin levels in obese and normal weight individuals with chronic periodontitis. J Indian Soc Periodontol.

[27] Kantarci A, Van Dyke TE (2005). Resolution of inflammation in periodontitis. J Periodontol.

[28] Gonçalves TE, Zimmermann GS, Figueiredo LC (2015). Local and serum levels of adipokines in patients with obesity after periodontal therapy: one-year follow-up. J Clin Periodontol.

[29] Gonçalves TE, Feres M, Zimmermann GS, Faveri M, Figueiredo LC, Braga PG, Duarte PM (2015). Effects of scaling and root planing on clinical response and serum levels of adipocytokines in patients with obesity and chronic periodontitis. J Periodontol.

[30] Devanoorkar A, Dwarakanath CD, Gundanavar G, Kathariya R, Patil SR (2012). Evaluation of serum resistin levels in periodontal health and disease and effects of non surgical periodontal therapy on its levels. Dis Markers.

[31] Gerber FA, Sahrmann P, Schmidlin OA, Heumann C, Beer JH, Schmidlin PR (2016). Influence of obesity on the outcome of non-surgical periodontal therapy - a systematic review. BMC Oral Health.

